# State-Dependent DNA Methylation Signatures Distinguish Acute from Stable Coronary Syndromes

**DOI:** 10.3390/ijms27052459

**Published:** 2026-03-07

**Authors:** Işık Tekin, Alten Oskay, Tülay Oskay, Murat Seyit, Mert Özen, Atakan Yılmaz, Yasemin Berberoğlu, Abdo A. Elfiky, Gergana Lengerova, Martina Bozhkova, Steliyan Petrov, İbrahim Türkçüer, Aylin Köseler

**Affiliations:** 1 Department of Cardiology, Faculty of Medicine, Pamukkale University, 20160 Denizli, Türkiye; itekin@pau.edu.tr; 2 Department of Emergency Medicine, Faculty of Medicine, Pamukkale University, 20160 Denizli, Türkiye; aoskay@pau.edu.tr (A.O.); mseyit@pau.edu.tr (M.S.); mert@pau.edu.tr (M.Ö.); atakany@pau.edu.tr (A.Y.); iturkcuer@pau.edu.tr (İ.T.); 3 Department of Cardiology, Denizli State Hospital, 20010 Denizli, Türkiye; oskaytulay@gmail.com; 4 Department of Biophysics, Faculty of Medicine, Pamukkale University, 20160 Denizli, Türkiye; yasemins@pau.edu.tr; 5 Department of Biophysics, Faculty of Science, Cairo University, Giza 12613, Egypt; abdo@sci.cu.edu.eg; 6 Department of Medical Microbiology and Immunology “Prof. Dr. Elissay Yanev”, Medical University of Plovdiv, 4002 Plovdiv, Bulgaria; gergana.lengerova@mu-plovdiv.bg (G.L.); martina.bozhkova@mu-plovdiv.bg (M.B.); steliyan.petrov@mu-plovdiv.bg (S.P.); 7 Research Institute, Medical University of Plovdiv, 4002 Plovdiv, Bulgaria

**Keywords:** DNA methylation, epigenetics, acute coronary syndrome, stable coronary syndrome, pathway analysis

## Abstract

Coronary artery disease presents heterogeneous clinical manifestations ranging from stable coronary syndrome (SCS) to acute coronary syndrome (ACS). Epigenetic mechanisms, particularly DNA methylation, may contribute to both chronic disease progression and acute plaque destabilization. However, genome-wide methylation differences between ACS, SCS, and healthy individuals remain incompletely characterized. Genome-wide DNA methylation analysis was performed in patients with ACS, patients with SCS, and healthy controls using pairwise comparisons (ACS vs. control, SCS vs. control, and ACS vs. SCS). Differentially methylated regions were identified using logistic regression implemented in the methylKit package in R. Regions with a false discovery rate-adjusted q-value < 0.05 and an absolute methylation difference (|Δβ|) > 20% were considered significant. Unsupervised hierarchical clustering revealed clear separation between ACS, SCS, and control samples, indicating distinct epigenetic profiles. ACS showed the most pronounced methylation alterations compared to controls, whereas SCS exhibited more moderate changes consistent with chronic epigenetic remodeling. Direct comparison between ACS and SCS identified dynamic, state-dependent methylation differences. Pathway analysis demonstrated enrichment of stress response, apoptotic signaling, and cell adhesion pathways in ACS, while SCS was primarily associated with pathways related to intercellular communication and vascular signaling. Our findings demonstrate that acute and stable coronary syndromes are characterized by distinct DNA methylation landscapes and pathway signatures. Epigenetic regulation of stress, adhesion, and signaling pathways may contribute to disease acuity and progression, highlighting DNA methylation as a potential molecular marker in coronary artery disease.

## 1. Introduction

Although important advances have been made in the prevention and treatment of coronary artery disease (CAD), it remains the leading cause of morbidity and mortality worldwide [1]. Although traditional risk factors such as dyslipidemia, hypertension, diabetes mellitus and smoking explain a large part of the disease burden, they do not fully account for the marked heterogeneity in clinical presentations and disease progression [2]. The biological mechanisms underlying particularly the transition from SCS to ACS have not yet been fully elucidated and thus, classical genetic and clinical risk models are clearly insufficient to meet the need for molecular-level insights [3]. There is increasing evidence that atherosclerosis today is a chronic inflammatory disease characterized by endothelial dysfunction, immune cell activation and maladaptive vascular remodeling. These processes are highly dynamic structures affected by various factors such as environmental exposures, metabolic status and aging. This dynamic feature suggests that the central role of regulatory mechanisms is modulating gene expression without altering the underlying DNA sequence. One of these mechanisms, epigenetic regulation, has emerged as a critical interface between genetic predisposition and environmental influences [4].

Epigenetics is a term referring to gene-regulating changes that can be inherited but also are potentially reversible and which arise independently from changes in the DNA sequence [5]. DNA methylation, which is the most common and in-depth studied epigenetic modification, involves the addition of a methyl group to cytosine residues—mostly at the CpG dinucleotides—and it plays a fundamental role in transcriptional control, chromatin organization and genomic stability [6]. Unlike genetic variants, DNA methylation patterns are tissue-specific, context-dependent, and sensitive to environmental stimuli; due to these features, they are of paramount importance in the pathogenesis of complex diseases, such as CAD [7]. Epigenome-wide association studies (EWASs) conducted over the last decade have found that DNA methylation loci are related to various cardiovascular risk factors such as inflammation, lipid metabolism, endothelial function, and immune cell differentiation [8]. Moreover, large-sample cohort studies have shown that DNA methylation signatures obtained from blood are associated with subclinical atherosclerosis and can predict future coronary events. These findings suggest that epigenetic changes may occur before the manifestation of clinically apparent disease [9,10]. Although DNA methylation-related evidence linking CAD is rapidly increasing, the number of studies focused on epigenetic differences between acute and stable clinical conditions is relatively limited. Acute coronary syndrome represents a pathological transition that is characterized by the destabilization of an atherosclerotic plaque, thrombosis development, and intensive inflammatory activation [11]. Experimental and clinical studies have revealed that cell adhesion molecules, calcium signaling, stress response pathways, and endothelial barrier disruption play a central role in triggering acute coronary events [12].

Recent epigenetic studies indicate that acute myocardial infarction and acute coronary syndrome are associated with widespread DNA methylation changes in peripheral blood cells; these changes are especially concentrated in genes related to immune activation, apoptosis, and stress signaling [13]. These observations suggest that epigenetic regulation can contribute not only to the development of chronic atherosclerotic load but also to the degree of acuteness of the disease. However, most of the current studies have been limited to comparing acute cases with healthy controls; they have not clearly aimed at distinguishing the epigenetic signatures specific to acute and stable coronary disease states. One of the most significant gaps in the current literature is the lack of integrated analyses that simultaneously and holistically compare DNA methylation profiles between patients with ACS, SCS, and healthy subjects. As long as a direct comparison between acute and stable disease conditions is not made, it will remain unclear whether the observed methylation changes simply reflect general atherosclerotic processes or are specifically related to mechanisms of plaque instability and acute ischemic events [14]. Moreover, a significant part of the previous EWASs has focused on single locus-level associations, thus limiting the biological interpretability of the findings. On the other hand, network-based and pathway-centric methods provide a complementary framework that helps to identify coordinated epigenetic changes (methylation) along crosstalking genes and signal pathways. It is increasingly recognized that such holistic and integrated strategies are becoming indispensable for understanding complex cardiovascular phenotypes [15].

In this research, we aimed to thoroughly characterize genome-wide DNA methylation patterns among three clinically significantly different groups comprising patients with acute coronary syndrome, patients with stable coronary syndrome, and healthy control individuals. To this end, we used robust statistical thresholds, unsupervised hierarchical clustering and network-based pathway enrichment analyses to identify state-dependent epigenetic signatures of disease and to develop a holistic epigenetic model of the progression of coronary artery disease. The findings obtained in line with these goals provide new insights into the epigenetic mechanisms underlying both chronic coronary disease and its acute clinical reflections and highlight DNA methylation as a potential molecular marker of the severity of the disease.

## 2. Results

### 2.1. Study Population and Sequencing Output

A total of 300 individuals were initially recruited for the study, including 100 patients with acute coronary syndrome (ACS), 100 patients with stable coronary syndrome (SCS), and 100 healthy controls. All participants provided peripheral blood samples for genome-wide DNA methylation analysis. Following library preparation and high-throughput sequencing, samples were subjected to quality control filtering based on DNA integrity, bisulfite conversion efficiency, sequencing depth, and minimum coverage criteria (≥10 reads per CpG site). After applying these predefined quality thresholds, 76 ACS samples, 62 SCS samples, and 100 control samples were retained for downstream differential methylation analysis. After filtering low-coverage CpG sites, a total of 442,354 CpG loci were included in the final genome-wide methylation analysis across all samples.

### 2.2. Global DNA Methylation Differences Between Groups

**Figure 1:**
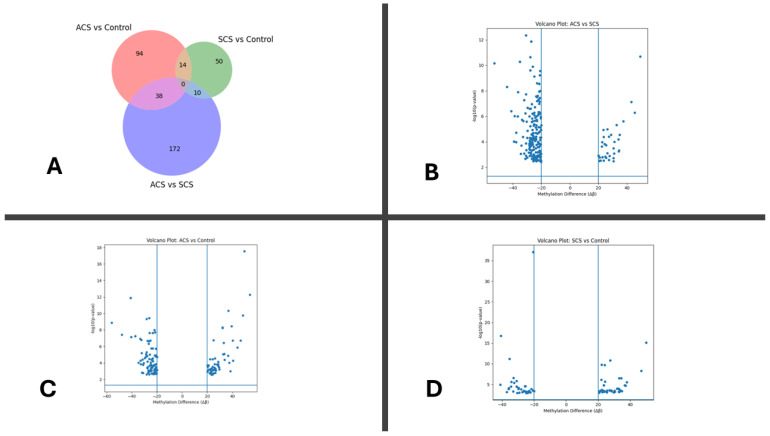
Genome-wide differential DNA methylation across acute and stable coronary syndromes. (**A**) Three-way Venn diagram illustrating the overlap of differentially methylated loci (DMLs; |Δβ| ≥ 20%; FDR-adjusted q < 0.05) across the following pairwise comparisons: ACS (n = 76) vs. control (n = 100), SCS (n = 62) vs. control (n = 100), and ACS (n = 76) vs. SCS (n = 62). A total of 116 DMLs were identified in the ACS vs. control comparison, 74 in the SCS vs. control comparison, and 220 in the ACS vs. SCS comparison. (**B**–**D**) Volcano plots displaying methylation effect size (Δβ) versus statistical significance (−log10 *p*-value) for each pairwise comparison: (**B**) ACS (n = 76) vs. control (n = 100), (**C**) SCS (n = 62) vs. control (n = 100), and (**D**) ACS (n = 76) vs. SCS (n = 62). Each point represents an individual CpG locus. Positive Δβ values indicate relative hypermethylation, whereas negative Δβ values indicate relative hypomethylation in the first group of each comparison. Vertical dashed lines indicate the |Δβ| ≥ 20% threshold. Statistical significance was assessed using logistic regression with FDR-adjusted q < 0.05; loci meeting both statistical and effect size thresholds were considered differentially methylated.

Genome-wide differential methylation analysis was performed across 442,354 CpG loci that passed quality control filtering. Pairwise comparisons were conducted between ACS (n = 76) and controls (n = 100), SCS (n = 62) and controls (n = 100), and ACS (n = 76) and SCS (n = 62) using logistic regression with FDR-adjusted q-values and an absolute methylation difference threshold of |Δβ| ≥ 20%. The highest number of differentially methylated loci (DMLs) was observed in the ACS vs. SCS comparison (n = 220), followed by the ACS vs. control comparison (n = 116), whereas the SCS vs. control comparison showed the lowest number of alterations (n = 74) (Figure 1A). These findings indicate that disease acuity is associated with substantial epigenetic divergence. Across comparisons, ACS-related analyses exhibited a predominance of hypermethylated loci, while the SCS vs. control comparison demonstrated a more balanced distribution of hyper- and hypomethylated loci. This pattern suggests increased epigenetic repression in the acute disease state relative to stable disease and healthy conditions. Genomic context analysis further revealed that the majority of DMLs were located in CpG shore and open sea regions rather than CpG islands, consistent with regulatory-region-associated methylation remodeling observed in complex cardiovascular phenotypes.

The overall number of differentially methylated loci identified in each comparison is summarized in Figure 1A. The ACS vs. SCS comparison exhibited the highest number of differentially methylated loci (n = 220), followed by the ACS vs. control comparison (n = 116), whereas the SCS vs. control comparison showed the lowest number of alterations (n = 74). Across comparisons, ACS-related analyses were characterized by a predominance of hypermethylated loci, while SCS demonstrated a more balanced distribution of hyper- and hypomethylated loci. CpG context analysis further indicated that most differentially methylated loci were located in CpG shore and open sea regions rather than CpG islands, consistent with regulatory-region-associated methylation remodeling.

### 2.3. Unsupervised Clustering Reveals State-Dependent Epigenetic Separation

**Figure 2:**
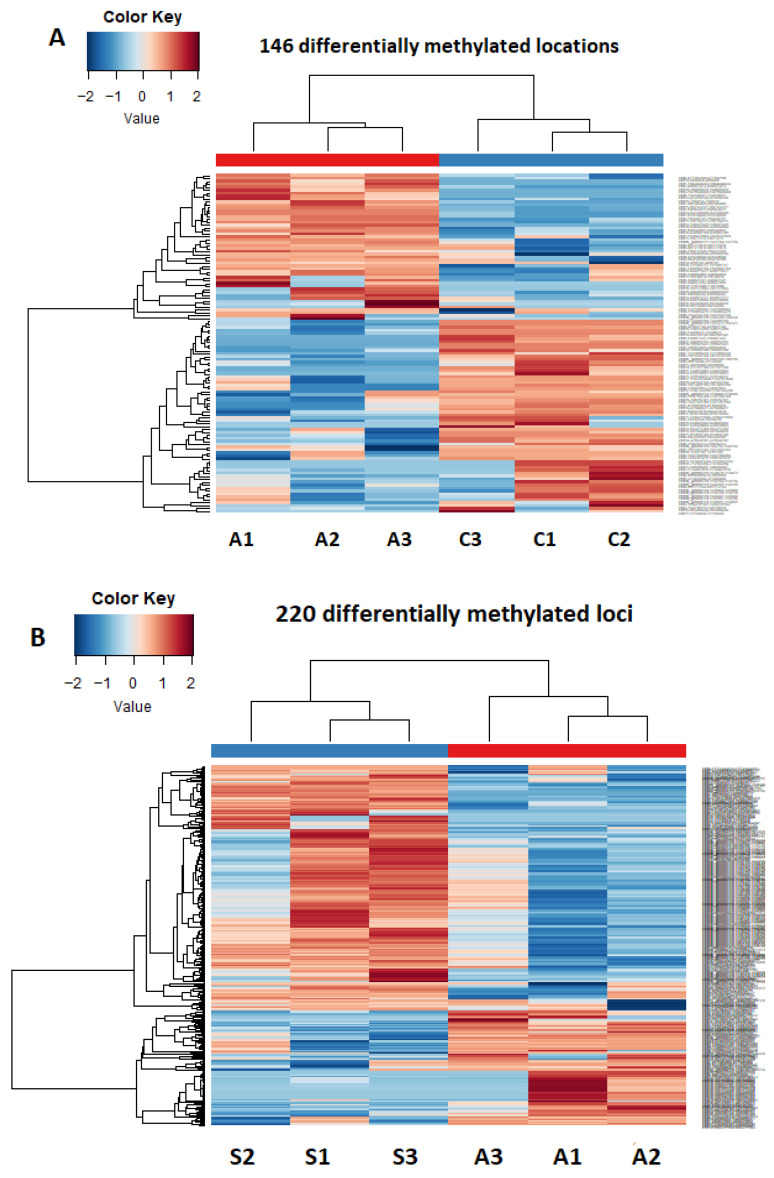

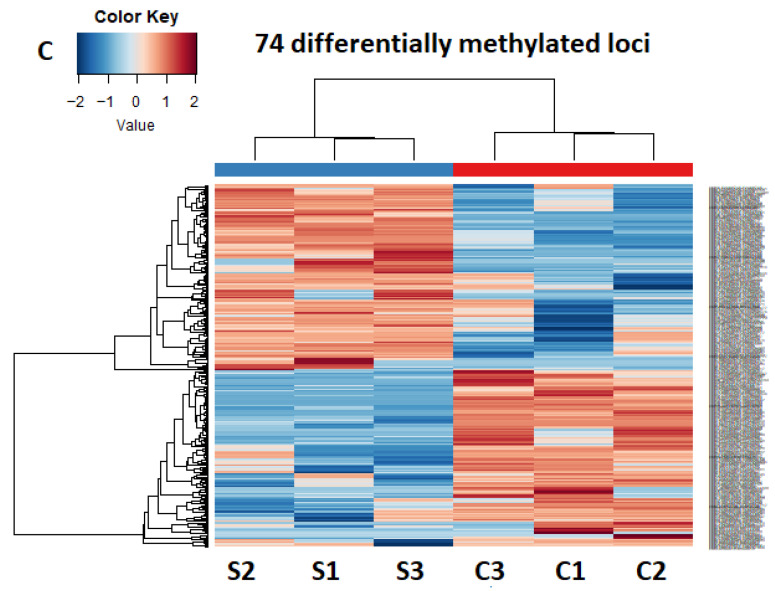
Unsupervised hierarchical clustering of differentially methylated loci across study groups. Columns represent individual samples included in the final quality-controlled methylation analysis (n = 76 ACS, n = 62 SCS, and n = 100 control). In the heatmaps, A denotes acute coronary syndrome (ACS) samples, S denotes stable coronary syndrome (SCS) samples, and C denotes control samples. (**A**) Heatmap showing 116 differentially methylated loci (|Δβ| ≥ 20%; FDR-adjusted q < 0.05) identified in the ACS (n = 76) versus control (n = 100) comparison (AvC). Unsupervised hierarchical clustering based on methylation levels demonstrates clear separation between ACS and control samples, indicating a distinct acute disease-associated epigenetic profile. (**B**) Heatmap of 220 differentially methylated loci identified in the ACS (n = 76) versus SCS (n = 62) comparison (AvS). Samples cluster according to disease acuity, highlighting methylation changes specifically associated with the acute phase rather than the mere presence of coronary artery disease. (**C**) Heatmap representing 74 differentially methylated loci identified in the SCS (n = 62) versus control (n = 100) comparison (SvC). Although overall methylation differences are less pronounced than in acute comparisons, hierarchical clustering reveals a consistent epigenetic signature associated with stable disease and chronic vascular remodeling. For all panels, rows represent differentially methylated CpG loci and columns represent individual samples. Methylation levels are displayed as standardized β values (Z-scores), where red indicates relative hypermethylation and blue indicates relative hypomethylation. Dendrograms were generated using Pearson correlation distance and complete linkage. For visual clarity, individual sample identifiers are not displayed; each column represents one individual sample.

Unsupervised hierarchical clustering was performed using significant differentially methylated loci (DMLs) identified in each pairwise comparison: ACS (n = 76) vs. control (n = 100), ACS (n = 76) vs. SCS (n = 62), and SCS (n = 62) vs. control (n = 100). To evaluate whether differentially methylated loci could distinguish clinical groups at the sample level, clustering was conducted using Pearson correlation-based distance and complete linkage. In the ACS vs. control comparison, clustering based on 116 significant DMLs resulted in clear separation between ACS and control samples (Figure 2A). ACS samples predominantly exhibited higher methylation levels relative to controls, indicating a robust acute disease-associated epigenetic signature. Direct comparison between ACS and SCS, based on 220 DMLs, demonstrated consistent but partial segregation of samples according to disease acuity (Figure 2B). This finding suggests dynamic and state-dependent epigenetic differences between acute and stable coronary syndromes. In the SCS vs. control comparison, clustering using 74 DMLs also separated SCS patients (n = 62) from controls (n = 100), although group distinction was less pronounced compared with ACS-related comparisons (Figure 2C). This pattern is consistent with more moderate but stable epigenetic remodeling associated with chronic disease.

In the SCS (n = 62) vs. control (n = 100) comparison, 74 differentially methylated loci (DMLs) separated SCS patients from healthy individuals (Figure 2C). Although group segregation was evident, methylation differences were less pronounced than those observed in ACS-related comparisons, consistent with chronic epigenetic remodeling rather than acute activation.

Distribution and direction of differentially methylated regions. The directionality of methylation changes across comparisons is shown in Figure 1B. ACS-related comparisons were characterized by a higher proportion of hypermethylated loci, whereas the SCS vs. control comparison exhibited a more even distribution of hyper- and hypomethylated regions. Genomic annotation revealed that the majority of differentially methylated loci were located within intronic and intergenic regions, while promoter-associated loci represented a smaller fraction. Despite their lower frequency, promoter-associated loci exhibited relatively larger methylation differences, suggesting potential regulatory relevance. CpG context analysis further demonstrated enrichment of methylation changes in CpG shores and open sea regions (Figure 1C).

### 2.4. Genomic Distribution and Annotation of Differentially Methylated Loci

Significant differentially methylated loci were further annotated according to genomic location and CpG context using hg19-based reference annotations. Across all pairwise comparisons, the majority of DMLs were located within intronic and intergenic regions, while promoter-associated loci represented a smaller fraction of significant sites. Despite their lower frequency, promoter-associated CpG sites generally exhibited larger absolute methylation differences, suggesting potential regulatory relevance. CpG context analysis revealed that most DMLs were enriched in CpG shores and open sea regions, rather than CpG islands. This distribution pattern is consistent with accumulating evidence that disease-associated methylation changes in complex phenotypes frequently occur in distal regulatory elements rather than canonical promoter CpG islands. Taken together, these findings indicate that epigenetic remodeling in coronary artery disease involves widespread regulatory-region methylation changes, with both promoter and non-promoter regions contributing to disease-associated signatures.

### 2.5. Comparison-Specific Differentially Methylated Genes

#### 2.5.1. ACS vs. Healthy Controls (AvC)

Comparison between ACS patients (n = 76) and healthy controls (n = 100) identified 116 significantly differentially methylated loci. Several loci demonstrated large effect sizes, with absolute methylation differences exceeding 50%. Notably, genes such as WFDC1, GRM5, and PRKG1 exhibited pronounced hypermethylation in ACS samples compared to controls. These genes are functionally associated with stress signaling, endothelial regulation, and inflammatory pathways, suggesting that acute coronary events are accompanied by robust epigenetic remodeling in stress-responsive and vascular signaling networks.

#### 2.5.2. SCS vs. Healthy Controls (SvC)

In the SCS (n = 62) vs. control (n = 100) comparison, 74 significant DMLs were identified. Differentially methylated loci were enriched in genes involved in vascular signaling, calcium-channel regulation, and intercellular communication. Representative genes included CACNA2D1, CACNA2D3, PRKCE, and MTHFS, suggesting that stable coronary syndrome is characterized by long-term epigenetic remodeling related to vascular tone regulation, metabolic adaptation, and chronic cellular signaling.

#### 2.5.3. ACS vs. SCS (AvS)

Direct comparison between ACS (n = 76) and SCS (n = 62) revealed 220 significant DMLs. These loci distinguished acute from stable disease states and were enriched in genes associated with cell adhesion, calcium signaling, and intracellular communication. Key differentially methylated genes included CDH4, CNTNAP2, NRXN3, NEGR1, PDE1A, and PDE1C. The observed methylation differences suggest dynamic epigenetic regulation linked to endothelial integrity, plaque instability, and calcium-dependent signaling pathways during acute coronary events.

### 2.6. Network-Based Pathway Enrichment Analysis

To identify coordinated biological processes associated with differential methylation, network-based pathway enrichment analysis was performed using the pathfindR framework. Active subnetworks were constructed based on significantly differentially methylated genes and assessed for pathway overrepresentation. In the ACS vs. control comparison, enriched pathways were predominantly related to stress-activated signaling and apoptotic processes, including MAPK-mediated stress response and apoptotic signaling pathways (Table 1). These findings align with the acute inflammatory and ischemic stress environment characteristic of acute coronary events.

Direct comparison between ACS and SCS revealed significant enrichment of cell adhesion molecule (CAM) pathways, calcium signaling pathways, and endothelial junction organization. These results suggest that epigenetic regulation of adhesion integrity and calcium-dependent intracellular signaling mechanisms may underlie plaque destabilization and disease acuity.

In contrast, the SCS vs. control comparison showed enrichment in pathways related to calcium-channel regulation, metabolic processes, and PKC-mediated signaling. This pattern is consistent with sustained vascular remodeling and metabolic adaptation associated with chronic coronary disease. Notably, a subset of genes involved in RNA metabolism and miRNA-mediated regulation (including NSUN6, ANKDD1A, MIR3648-1, and MIR3687-1) demonstrated consistent differential methylation across all comparisons, suggesting the presence of a shared core epigenetic signature of coronary artery disease independent of clinical state.

## 3. Discussion

In the present study, we performed a comprehensive genome-wide DNA methylation analysis to delineate epigenetic differences among ACS, SCS, and healthy control subjects. By integrating differential methylation analysis, unsupervised clustering, and network-based pathway enrichment, we demonstrated that coronary artery disease is characterized by both shared and state-dependent epigenetic alterations. Our findings support a layered epigenetic model in which chronic methylation changes associated with stable disease create a permissive background, upon which additional, dynamic alterations contribute to acute coronary events.

Epigenetic distinction between acute and stable coronary syndromes: Hierarchical clustering analysis based on differentially methylated loci without supervision revealed that the ACS, SCS, and control groups were distinct from each other. While ACS samples showed the most prominent methylation changes, SCS samples revealed more moderate but consistent epigenetic changes. These findings are compatible with recent epigenome-wide association studies (EWASs) that demonstrated that peripheral blood DNA methylation profiles can distinguish individuals with coronary artery disease and predict future acute coronary events [16].

Significantly, comparisons associated with ACS exhibited a pattern of predominance of hypermethylation. This indicates widespread epigenetic repression or reprogramming during acute ischemic and inflammatory stress. Similarly, recent studies on acute myocardial infarction and adverse cardiovascular outcomes have also reported epigenetic signatures dominated by hypermethylation [10,13]. On the other hand, a more balanced distribution of hypermethylated and hypomethylated loci observed in the SCS group may reflect long-term adaptive epigenetic changes associated with chronic vascular remodeling.

Genomic context and regulatory implications of differential methylation: Consistent with the up-to-date cardiovascular epigenetics literature, most of the differentially methylated loci defined by our study were located not in CpG islands but in CpG shore and open sea regions, particularly in intronic and intergenic regions. More and more pieces of evidence indicate that DNA methylation alterations associated with the disease are most of the time distal regulatory elements and enhancer regions and that gene expression is affected by binding-specific (context-dependent) mechanisms rather than by the classical promoter-silencing mechanisms. This corresponds to the findings revealed by multi-cohort epigenome-wide studies which show that methylation signals associated with the incidence of cardiovascular disease are largely concentrated in non-promoter and regulatory regions [17].

Promoter-associated loci, while constituting a smaller portion of the differentially methylated regions, displayed higher methylation effect sizes, thus emphasizing their potential functional importance. Taken together, these findings suggest a model in which subtle but coordinated epigenetic changes occurring broadly at regulatory regions contribute to the phenotypes of coronary artery disease. When considered together, the findings of this study demonstrate that the phenotypes of coronary artery disease are shaped not only by single and large epigenetic changes but also by the cumulative effect of subtle but coordinated epigenetic rearrangements occurring at promoters, enhancers, and other regulatory regions. This approach is also consistent with the current literature that shows in multi-cohort epigenome-wide analyses that methylation signals associated with cardiovascular disease incidence reside in both high-effect promoter regions and distal regulatory elements together [17].

Gene-level insights into acute and chronic disease mechanisms: Beyond enrichment analyses at the gene level, some of the differentially methylated genes (DMGs) described in this study are in fact well-known or emerging players in cardiovascular biology. For instance, hypermethylation of stress and cellular signaling genes such as PRKG1 (Protein Kinase, cGMP-Dependent, Type I) and GRM5 (Glutamate Metabotropic Receptor 5) observed in the comparison of the ACS vs. control groups can be interpreted as the endothelial function, platelet signaling and inflammation pathways being epigenetically reprogrammed. It has indeed been demonstrated in recent studies that disruption of PRKG1 signaling is associated with endothelial dysfunction, loss of vascular reactivity and development of atherosclerosis [18]. Also, it has been reported that the inflammatory cell activation, oxidative stress response, and vascular inflammation processes mediated by GRM5 signaling can play a role in the pathogenesis of cardiovascular diseases [19].

Systemic inflammation, especially inflammatory conditions associated with markers such as C-reactive protein, has been found to lead to a partly joint epigenetic remodeling in the majority of cardiovascular and inflammatory diseases. This phenomenon is often referred to in the literature as “epigenetic drift” due to immune activation [20,21]. Nevertheless, it is expected that differences in methylation between acute and chronic disease conditions would, in part, reflect the general inflammatory response. Studies on ACS, however, have identified methylation regions that are reproducible and disease-specific and cannot be reduced to mere general inflammatory signaling. For instance, loci such as cg03609847 (PIGG) and cg16749093 (EHBP1L1) have been found to be associated with ACS independently of systemic inflammation markers [13]. In addition, the fact that these changes have been observed not only in peripheral blood but also directly in atherosclerotic plaque tissue implies that these epigenetic differences may not be just a systemic reaction but a direct part of coronary pathogenesis [13].

Specificity is a feature that stands out even when comparing ACS with other acute-chronic disease models. For example, epigenome-wide association studies in chronic kidney disease mostly identify CpG regions related to renal function indicators such as eGFR and albuminuria [22]. Although there are common “cardio-renal-metabolic” pathways, the CpG regions specific to renal damage differ from the loci associated with myocardial infarction or acute coronary events [20,22]. It shows that inflammatory components are shared, but the biological context of the disease determines the epigenetic target regions. There are factors that make ACS epigenetically even more specific. Increased mitochondrial DNA methylation in ACS patients compared to stable coronary artery patients suggests a role for metabolic stress and mitochondrial dysfunction in acute events [23]. Also, the ability of 5-hydroxymethylation (5hmC)-based multi-marker panels to distinguish healthy controls, stable coronary artery patients, and acute myocardial infarction cases with high accuracy (AUC ≈ 0.9) supports the fact that ACS is not simply a state of more severe inflammation but a biologically different phase [24]. Some loci such as FKBP5 being associated with the heart damage and recovery processes at the acute phase also indicate that epigenetic regulation may be linked to functional cardiac outcomes [9].

In line with this literature, in our study, a comparison between AMI and SCS revealed the highest number of different methyl loci (n = 220), which shows that the severity and the level of acuity of the disease are related to a marked epigenetic differentiation. Furthermore, the dominance of hypermethylation in AMI-associated comparisons provides a pattern compatible with increased epigenetic repression and immune activation in the acute phase. However, the fact that the CpG regions are mainly localized in shore and open sea regions suggests that these changes may not be promoter-focused classical inflammation signaling but rather fine-tuned gene regulation in regulatory regions. Hence, our findings not only partially reflect a general acute inflammatory response but also reveal the epigenetic signatures that are associated with vascular integrity, cellular adhesion, and metabolic stress mechanisms, which are the hallmarks of the acute phase of coronary disease. On the other hand, it should be kept in mind that peripheral blood methylation profiles can be influenced by changes in leukocyte composition in the acute phase and future studies should be performed with cell type-specific analyses to evaluate this specificity in more detail.

The direct comparison of ACS and SCS groups through ACS has revealed the presence of differential DNA methylation in genes related to cell adhesion and calcium signaling such as CDH4 (Cadherin 4), CNTNAP2 (Contactin-Associated Protein-Like 2), NRXN3 (Neurexin 3) and NEGR1 (Neuronal Growth Regulator 1). Cadherins are calcium-dependent adhesion molecules that play a critical role in maintaining endothelial integrity, and adhesion signalization disruption is one of the fundamental mechanisms behind atherosclerotic plaque instability and rupture. Therefore, the epigenetic modulation of the adhesion genes CDH4 and related genes might contribute to the acute destabilization of atherosclerotic plaques. Similarly, the differential methylation observed in calcium-related signaling genes such as PDE1A (Phosphodiesterase 1A) and PDE1C (Phosphodiesterase 1C) highlights the role of calcium-dependent intracellular signaling in acute coronary events.

In the comparison of SCS with the control group, it was found that differentially methylated genes were enriched in chronic vascular signaling and metabolic regulation pathways. Differential methylation detected in genes such as CACNA2D1 (Calcium Voltage-Gated Channel Auxiliary Subunit Alpha2delta1) and CACNA2D3 (Calcium Voltage-Gated Channel Auxiliary Subunit Alpha2delta3), which encode auxiliary subunits of voltage-dependent calcium channels, points to long-term epigenetic regulation of calcium homeostasis and vascular smooth muscle function in stable disease state. In addition, methylation changes in genes like MTHFS (5,10-methenyltetrahydrofolate synthetase), DGKB (Diacylglycerol Kinase Beta) and PRKCE (Protein Kinase C Epsilon) that are involved in metabolic and phosphoinositide signaling suggest epigenetic adaptations that have been continuous rather than acute stress responses. CACNA2D1 and CACNA2D3 encode the α2δ auxiliary subunits of voltage-dependent calcium channels and play a crucial role in the stability of the calcium channel in the plasma membrane, the efficiency of the conduction, and the pharmacological sensitivity [25]. Since calcium entry in vascular smooth muscle cells directly determines vessel tone and contractility, the epigenetic regulation of these genes may be related to chronic vascular function adaptations. Differential methylation observed in SCS points to a long-term resetting of calcium homeostasis rather than an acute response.

MTHFS is a gene from carbon metabolism that is very important for folate metabolism and balance of methyl donors [26]. Cardiovascular diseases and especially ACS are closely associated with epigenetic modifications including DNA methylation [5,27,28,29,30]. Metabolic changes can modify the risk of CVD through modulating gene expression via epigenetic markers [31,32]. Studies on ACS patients have revealed that there are significant changes in the genome-wide DNA methylome [33]. These changes may affect many genes that are related to heart function and disease pathogenesis [15,34,35]. For instance, DNA methylation profiles of the genes linked to folate metabolism were associated with myocardial infarction risk [36]. Within the context of SCS, alterations in MTHFS methylation might be indicative of epigenetic adaptations developed under chronic metabolic stress. Therefore, the epigenetic regulation of MTHFS under metabolic stress and its linkage to chronic vascular adaptations such as ACS have a solid rationale within the framework of folate metabolism and epigenetic regulation. This gene’s critical role in the mechanisms of cellular metabolism status and methylation capacity adaptation truly supports our thought that it might contribute to the long-term adaptations of the cardiovascular system.

The diacylglycerol kinase family members regulate various cellular processes by converting major signaling molecules such as diacylglycerol and phosphatidic acid. These include development, cell division and proliferation, neuronal and immune responses, vascular traffic, and apoptosis [37]. It is widely accepted that DGKB (Diacylglycerol Kinase Beta) regulates phosphoinositide signaling by converting diacylglycerol to phosphatidic acid and that this pathway plays a key role in cell proliferation, metabolism, and vascular cell response [37,38,39,40,41,42]. However, from the literature review conducted, it was found that it is difficult to find direct and recent scientific evidence on the differential methylation of the DGKB gene as a continuous but low-level cellular activation of epigenetic correspondences in the case of myocardial infarction. The epigenetic regulations of other members of the DGK family (e.g., DGKα) have been suggested to play a role in different pathologies such as radiation-induced fibrosis [43]. Although it was demonstrated that calcium signaling and phosphatidylinositol signaling are affected by the hypermethylation of the gene body in the diabetic heart [44], this finding is not specific to DGKB or directly related to myocardial infarction. Therefore, more studies are needed on the epigenetic role of DGKB in myocardial infarction.

PRKCE (Protein kinase C epsilon) is one of the members of the protein kinase C family and in cardiomyocytes and vascular cells, it is related to cardioprotective signaling pathways, metabolic adaptation and stress tolerance [45,46,47,48,49]. PKCε is known to protect the heart from ischemia and reperfusion injury and to mediate the effects of ischemic preconditioning [50,51,52,53,54,55]. There are several different epigenetic changes in the PRKCE gene that may well be related to the later roles of the gene in chronic adaptations and cell survival mechanisms rather than acute injury. This has been widely corroborated in the literature. Studies have indicated that downregulation of PKCε gene expression through promoter methylation has negative effects on the cardiac resistance to ischemic/reperfusion (I/R) injury [50,52,53,56,57]. For instance, prenatal nicotine or cocaine exposure may epigenetically reduce PKCε gene expression and thereby elevate adult cardiac sensitivity [50,52,53,56,57]. Such epigenetic modifications change the heart’s capacity to adapt long-term when it is faced with stress. Moreover, it has also been found that in acute myocardial infarction patients, PKCε expression in platelets increased during the acute event and then returned to normal levels; thus, PKCε could play the role of a marker of a peri-infarction period [58,59]. These data indicate that PRKCE plays an important role in both the acute response and the long-term adaptation processes. Changes in the methylation status of this gene suggest that in stable disease, rather than acute injury, long-term cellular adaptation and survival mechanisms are predominant.

Core epigenetic signature of coronary artery disease: NSUN6 (NOP2/Sun RNA Methyltransferase Family Member 6), ANKDD1A (Ankyrin Repeat and Death Domain-Containing 1A), MIR3648-1 (MicroRNA 3648-1), MIR3687-1 (MicroRNA 3687-1), and LOC100507412 (Uncharacterized LOC100507412) genes, which were differentially methylated in a consistent manner in all comparisons, point to the existence of a core epigenetic signature of coronary artery disease independent of clinical presentation. Members of the NSUN family, especially NSUN2, NSUN3, NSUN4, and NSUN6, show changes in their expression as a response to oxidative stress, e.g., arsenite [60]. It has been demonstrated that NSUN2-mediated RNA methylation is a crucial mechanism in reprogramming protein synthesis and the stress resistance of cells under stress [60]. This situation indicates that the NSUN family is involved in cellular adaptation mechanisms. In cardiovascular diseases, such as cardiomyopathy and heart failure, the role of m5C modifications in mitochondrial mRNAs was examined. It has been suggested that cardiac-specific inactivation of NSUN4 leads to cardiomyopathy and affects mitochondrial translation and respiratory chain functions [61,62,63,64]. These data highlight the crucial role of m5C methyltransferases in the regulation of protein synthesis and cellular functions in heart tissue. It was discovered that NSUN2 attenuates doxorubicin-induced myocardial injury by enhancing the Nrf2-mediated antioxidant stress response and that Nrf2 mRNA undergoes m5C modification [64]. This indicates that members of the NSUN family can influence cardiac functions through protein synthesis and cellular adaptation under stress conditions. The differential methylation of this gene suggests that the protein synthesis and cellular adaptation mechanisms in coronary artery disease could be epigenetically reprogrammed.

The association of ANKDD1A with hypoxia-responsive cellular mechanisms and the adaptive mechanisms that arise under oxygen deprivation conditions is a theoretically supported hypothesis. Specifically, ANKDD1A has been characterized as a functional tumor suppressor gene in a hypoxia microenvironment. In glioblastoma cells, hypermethylation of ANKDD1A leads to a decrease in gene expression. ANKDD1A interacts directly with HIF1AN (Hypoxia-Inducible Factor 1 Alpha Inhibitor) to inhibit the transcriptional activity of HIF1α and to reduce the stability of HIF1α [65]. This mechanism clearly indicates that ANKDD1A regulates the processes of cellular adaptation and responses to hypoxia through methylation changes. Epigenetic regulations, especially DNA methylation, play a key role in myocardial ischemia and reperfusion [66]. Furthermore, an increase in the activity of DNA methyltransferases in chronic hypoxia has been shown to lead to cardiac fibrosis [67]. These findings corroborate the general framework that hypoxic stress may lead to epigenetic changes and tissue adaptations; hence, consistent methylation changes in ANKDD1A might be a common epigenetic signature of chronic ischemia and tissue hypoxia in both stable and acute disease conditions.

From the recent literature review, direct and detailed scientific evidence of MIR3648-1, MIR3687-1 and LOC100507412 gene-specific differential methylations in coronary artery disease or their functional consequences is not available. Although the general role of microRNAs and other non-coding RNAs in the epigenetic regulation of cardiovascular diseases is well-known, direct supportive findings for these specific genes are not available [67,68,69]. The consistent methylation changes at the LOC100507412 locus, the function of which has not been fully elucidated, indicate the presence of new and as of yet undefined epigenetic regulators that may have a role in the pathogenesis of coronary artery disease and, thus, represent potential functional candidates for future studies. The fact that these genes were differentially methylated in the same manner in all the comparisons indicates that the epigenetic changes we identified may be not only the transient events related to the disease acuteness but also stable and state-independent epigenetic mechanisms reflecting the essential biological background of coronary artery disease.

Integrated pathway-based epigenetic model: The schematic integrates gene-level differential DNA methylation with network-based pathway enrichment results across clinical comparisons. In the AvC (ACS vs. control) comparison, hypermethylation of stress- and signaling-related genes (PRKG1, GRM5, and ARHGEF12) and hypomethylation of TIAM1 (T-cell lymphoma invasion and metastasis 1) are highlighted, consistent with acute inflammatory and ischemic responses. In a study published in 2024, it was identified that methylation in the ARHGEF12 gene (especially the cg00395063 probe) was statistically significantly associated with the risk of CAD and myocardial infarction and was correlated with the increase in this risk [70]. This gene has also been linked with major risk factors for CAD such as hypertension [70]. Although in the methylation studies of ACS, PRKG1 is usually not mentioned as the “top hit” (the topmost) gene alone, it is the backbone of “Vascular Smooth Muscle Contraction” and cGMP-PKG Signaling Pathway, which play a key role in the pathogenesis of ACS [5,71]. These pathways are among the main biological processes where the differentially methylated regions detected in ACS patients are enriched [5].

**Figure 3:**
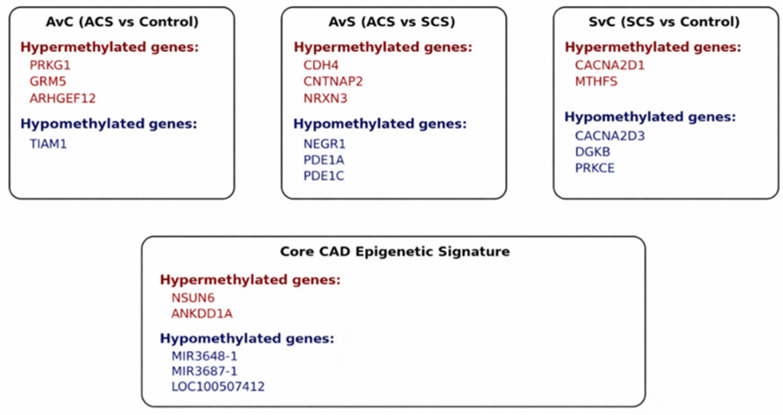
Gene-annotated DNA methylation model of coronary artery disease (red: hypermethylation; blue: hypomethylation).

GRM5 has been reported in AKS-associated methylation network analyses and especially in calcium signaling pathway enrichment [5,72]. While AKS methylation studies (e.g., the work of Long et al.) have highlighted genes such as PRKCZ or EHBP1L1 as main markers, GRM5 is one of the genes regulating calcium flow in the cardiovascular system [10,13]. The AvS (ACS vs. SCS) comparison emphasizes acuity-specific regulation of cell adhesion and calcium-linked signaling pathways, characterized by hypermethylation of CDH4, CNTNAP2, and NRXN3 and hypomethylation of NEGR1, PDE1A, and PDE1C, implicating mechanisms related to plaque instability and endothelial dysfunction. In contrast, the SvC (SCS vs. control) comparison reflects chronic disease-associated epigenetic signatures involving calcium-channel regulation and metabolic pathways, with hypermethylation of CACNA2D1 and MTHFS and hypomethylation of CACNA2D3, DGKB, and PRKCE, consistent with long-term vascular adaptation. The bottom panel depicts a core CAD epigenetic signature shared across all comparisons, comprising hypermethylated (NSUN6 and ANKDD1A) and hypomethylated (MIR3648-1, MIR3687-1, and LOC100507412) loci, suggesting common epigenetic mechanisms underlying disease susceptibility independent of clinical presentation (Figure 3).

By integrating genome-wide methylation changes with network-based pathway enrichment analyses, we derived a comprehensive epigenetic model of the progression of coronary artery disease (Figure 3). According to this model, stable coronary syndrome is characterized by chronic and sustained epigenetic remodeling of vascular signaling, metabolic pathways and intercellular communication. On top of this epigenetic landscape, stress response, changes in cell adhesion and the calcium-dependent signaling pathway influenced by dynamic methylation changes, and the transition from stable disease to acute coronary syndrome play a determining role. This comprehensive approach reveals that changes in DNA methylation are closely associated not only with the presence of coronary artery disease but also with the severity of the disease. Therefore, the proposed model provides a mechanistic framework for how epigenetic regulation may shape the continuum from chronic vascular remodeling to acute plaque destabilization and positions DNA methylation as a potential biomarker and risk stratification tool in coronary artery disease.

## 4. Materials and Methods

### 4.1. Study Design and Sample Groups

This study was designed as a cross-sectional epigenome-wide association study including three groups: acute coronary syndrome (ACS), stable coronary syndrome (SCS), and healthy controls. Initially, 100 individuals were recruited for each group (total n = 300). Genome-wide DNA methylation sequencing was performed only on samples that met predefined quality control criteria, including DNA integrity, bisulfite conversion efficiency, successful library preparation, and minimum sequencing coverage (≥10 reads per CpG site). After quality control filtering, 76 ACS samples, 62 SCS samples, and 100 control samples were retained for the final differential methylation analysis. All downstream bioinformatic and statistical analyses were conducted using these quality-controlled samples. Patients with ACS and SCS were recruited from individuals who underwent coronary angiography at the Pamukkale University Cardiology Clinic, while the control group consisted of apparently healthy individuals with no history or clinical evidence of coronary artery disease. The mean age of the study population was 55 years (range: 48–68 years), and the proportion of female and male participants was equal within each group. DNA methylation profiles were compared using pairwise analyses between ACS and healthy controls (AvC), SCS and healthy controls (SvC), and ACS and SCS (AvS). All analyses were conducted separately for each comparison to identify disease-specific and state-dependent epigenetic alterations. This study was conducted in accordance with the Declaration of Helsinki and approved by the Institutional Review Board (or Ethics Committee) of Pamukkale University (E-60116787-020-49148, date 11 July 2019).

### 4.2. Sample Collection and DNA Isolation

Peripheral blood samples were collected from patients with ACS, patients with stable coronary syndrome, and healthy control subjects according to standard clinical procedures. Genomic DNA was isolated from whole blood samples using the QIAamp DNA Blood Mini Kit (QIAGEN, Hilden, Germany), following the manufacturer’s instructions. DNA concentration and purity were assessed using spectrophotometric measurements, and samples with sufficient quality were used for downstream analyses.

### 4.3. Bisulfite Conversion of Genomic DNA

Genomic DNA samples were subjected to sodium bisulfite treatment to convert unmethylated cytosines into uracils while leaving methylated cytosines unchanged. Bisulfite conversion was performed using the EZ DNA Methylation-Gold™ Kit (Zymo Research, Irvine, CA, USA) according to the manufacturer’s protocol. Converted DNA samples were subsequently purified and stored at −20 °C until further processing.

### 4.4. Genome-Wide DNA Methylation Profiling

Following bisulfite conversion, genome-wide DNA methylation profiling was performed using a high-throughput sequencing-based approach. Sequencing libraries were prepared from bisulfite-converted DNA using the NEBNext^®^ Enzymatic Methyl-seq Kit (New England Biolabs, Ipswich, MA, USA) according to the manufacturer’s instructions. Libraries were sequenced on an Illumina NextSeq platform (Illumina, San Diego, CA, USA) using paired-end sequencing. Methylation coverage files generated from sequencing data were used as input for downstream bioinformatic analyses.

### 4.5. Quality Control of Methylation Data

Quality control procedures were applied to ensure reliable methylation measurements. CpG sites with low sequencing coverage were excluded, and only loci with a minimum read depth of ≥10 reads per CpG site in each sample were retained for differential methylation analysis. These quality control steps ensured the robustness and reproducibility of methylation estimates across samples.

### 4.6. DNA Methylation Data Processing

Genome-wide DNA methylation analyses were performed in the R statistical environment (version 3.5.1). Methylation coverage files generated from sequencing data were imported into R as methylKit objects using a custom import function. Only CpG sites were included in the analysis. To ensure data quality, CpG sites with a sequencing read coverage of fewer than 10 reads in any sample were excluded from downstream analyses. After filtering, methylation levels were calculated as the percentage of methylated cytosines at each CpG site.

### 4.7. Exploratory Data Analysis

Exploratory analyses were conducted to assess sample similarity and overall methylation patterns. Pearson correlation coefficient matrices were generated based on methylation percentage values across samples. Unsupervised hierarchical clustering was performed using Pearson correlation-based distance and complete linkage to visualize relationships between samples and to evaluate group-level clustering. Heatmaps were generated to illustrate clustering patterns of differentially methylated loci.

### 4.8. Differential Methylation Analysis

Differentially methylated regions were identified using logistic regression models implemented in the methylKit package. For each pairwise comparison, logistic regression was applied to test for differences in methylation proportions between groups at individual CpG sites. Raw *p*-values obtained from logistic regression were adjusted for multiple testing using the SLIM (Sliding Linear Model) method to estimate false discovery rates (FDRs). CpG sites and regions with an adjusted q-value < 0.05 and an absolute methylation difference (|Δβ|) greater than 20% were considered statistically significant and retained for downstream analyses.

### 4.9. Genomic Annotation of Differentially Methylated Regions

Significant differentially methylated regions were annotated to genomic features using the GenomicRanges (GRanges) package in the R statistical environment. Annotation was performed against the human reference genome hg19. Each region was classified according to its genomic context, including promoter, exon, intron, and intergenic regions, as well as CpG context categories comprising CpG islands, shores, shelves, and open sea regions.

### 4.10. Pathway and Network-Based Enrichment Analysis

Functional pathway enrichment analyses were conducted using the pathfindR package in the R statistical environment. This network-based approach integrates differential methylation results with protein–protein interaction networks to identify biologically relevant active subnetworks. Active subnetworks were identified using a greedy search algorithm, which iteratively detects subnetworks enriched with differentially methylated genes. To account for the stochastic nature of the greedy algorithm, the analysis was repeated 10 times, and consistently enriched pathways were reported. Pathway enrichment analysis was performed using a one-sided hypergeometric test, restricted to genes within statistically significant active subnetworks. Resulting *p*-values were adjusted using the Bonferroni correction.

### 4.11. Statistical Considerations

All statistical analyses were conducted within the R statistical environment. Adjusted *p*-values were used to control for multiple testing where applicable. Results were visualized using heatmaps, hierarchical clustering dendrograms, and summary plots to facilitate interpretation of epigenetic differences across disease states.

## 5. Conclusions

Clinical implications and future directions: Recent studies suggest that DNA methylation signatures derived from blood can be used as biomarkers in cardiovascular risk classification and prognosis prediction [13,16]. The results of our study go one step further by showing that DNA methylation profiles can discriminate not only the presence of disease but also the severity level of the disease. This finding points to the potential of epigenetic markers to be used in identifying patients with stable coronary disease who are at higher risk of acute events.

However, there are some limitations to our study. The relatively limited sample size necessitates the confirmation of findings in broader and independent cohorts. Also, methylation changes have not been functionally validated at the transcriptomic or proteomic level. Future studies involving multi-omics approaches and longitudinal sampling are expected to elucidate more clearly the causal role of epigenetic changes in the progression of coronary artery disease.

In conclusion, this study reveals that acute and stable coronary syndromes are associated with DNA methylation patterns that have overlapping but also distinguishable features. While the stable disease condition is characterized by chronic epigenetic remodeling, acute coronary events are distinguished by stress response, cell adhesion, and calcium signaling pathways affecting condition-specific additional methylation changes. These findings provide new perspectives on the epigenetic mechanisms underlying coronary artery disease and highlight DNA methylation as a potential molecular marker of disease acuity. From a clinical point of view, it is believed that blood-based DNA methylation profiles could facilitate the identification of subgroups at risk for acute events among individuals with stable coronary disease and thus contribute to the development of future personalized risk stratification strategies.

## Figures and Tables

**Table 1:** Network-based pathway enrichment results for pairwise DNA methylation comparisons.

Comparison	Enriched Pathway (Database)	Representative Genes in Active Subnetworks	Direction of Methylation Trend	Adjusted *p*-Value
ACS vs. Control (AvC)	Stress-activated signaling pathways (KEGG)	PRKG1, GRM5, ARHGEF12	Predominantly hypermethylated	<0.01
	Apoptotic signaling pathways (REACTOME)	PRKG1, ARHGEF12	Hypermethylated	<0.05
	MAPK-mediated stress response (KEGG)	GRM5, TIAM1	Mixed (hyper/hypo)	<0.05
ACS vs. SCS (AvS)	Cell adhesion molecules (CAMs) (KEGG)	CDH4, CNTNAP2, NRXN3	Predominantly hypermethylated	<0.01
	Calcium signaling pathway (KEGG)	PDE1A, PDE1C, NEGR1	Predominantly hypomethylated	<0.05
	Endothelial junction organization (REACTOME)	CDH4, NEGR1	Mixed	<0.05
SCS vs. Control (SvC)	Calcium channel regulation (KEGG)	CACNA2D1, CACNA2D3	Mixed	<0.01
	Metabolic and folate-related pathways (REACTOME)	MTHFS, DGKB	Predominantly hypermethylated	<0.05
	PKC signaling and vascular adaptation (KEGG)	PRKCE, DGKB	Predominantly hypomethylated	<0.05
All comparisons (core signature)	RNA metabolism and epigenetic regulation (REACTOME)	NSUN6, ANKDD1A	Hypermethylated	<0.05
	miRNA-mediated gene silencing (KEGG)	MIR3648-1, MIR3687-1	Hypomethylated	<0.05

## Data Availability

The genome-wide DNA methylation sequencing dataset generated in this study has been submitted to the Gene Expression Omnibus (GEO) repository. The accession number will be provided upon final approval and release by GEO. The data will be made publicly available upon publication.

## Abbreviations

The following abbreviations are used in this manuscript:

ACS	Acute Coronary Syndrome
ANKDD1A	Ankyrin Repeat and Death Domain-Containing 1A
AvC	Acute Coronary Syndrome vs. Control
AvS	Acute Coronary Syndrome vs. Stable Coronary Syndrome
CAD	Coronary Artery Disease
CAMs	Cell Adhesion Molecules
CpG	Cytosine–Phosphate–Guanine
DMGs	Differentially Methylated Genes
DMRs	Differentially Methylated Regions
DNA	Deoxyribonucleic Acid
DGKB	Diacylglycerol Kinase Beta
EWAS	Epigenome-Wide Association Study
FDR	False Discovery Rate
FIH1 (HIF1AN)	Hypoxia-Inducible Factor 1 Alpha Inhibitor
GRM5	Glutamate Metabotropic Receptor 5
HIF1α	Hypoxia-Inducible Factor 1 Alpha
I/R	Ischemia/Reperfusion
KEGG	Kyoto Encyclopedia of Genes and Genomes
LOC	Locus
MAPK	Mitogen-Activated Protein Kinase
miRNA	MicroRNA
MIR3648-1	MicroRNA 3648-1
MIR3687-1	MicroRNA 3687-1
MTHFS	5,10-Methenyltetrahydrofolate Synthetase
NSUN6	NOP2/Sun RNA Methyltransferase Family Member 6
PDE1A	Phosphodiesterase 1A
PDE1C	Phosphodiesterase 1C
PKC	Protein Kinase C
PRKG1	Protein Kinase, cGMP-Dependent, Type I
PRKCE	Protein Kinase C Epsilon
q-value	FDR-Adjusted *p*-Value
RNA	Ribonucleic Acid
SCS	Stable Coronary Syndrome
SLIM	Sliding Linear Model
SvC	Stable Coronary Syndrome vs. Control
TIAM1	T-Cell Lymphoma Invasion and Metastasis 1
Δβ	Absolute Difference in DNA Methylation Level
